# Bioengineered *Matricaria recutita* Extract-Assisted Palladium Nanoparticles for the Congo Red Dye Degradation and Catalytic Reduction of 4-Nitrophenol to 4-Aminophenol

**DOI:** 10.3390/toxics9050103

**Published:** 2021-05-04

**Authors:** Maqsood Ahmad Malik, Abdulmohsen Ali Alshehri, May Abdullah Abomuti, Ekram Y. Danish, Rajan Patel

**Affiliations:** 1 Chemistry Department, Faculty of Sciences, King Abdulaziz University, P.O. Box 80203, Jeddah 21589, Saudi Arabia; aayalshehri@kau.edu.sa (A.A.A.); maabumoati@su.edu.sa (M.A.A.); eydanish@kau.edu.sa (E.Y.D.); 2 Biophysical Chemistry Laboratory, Centre for Interdisciplinary Research in Basic Sciences, Jamia Millia Islamia, New Delhi 110025, India; rajanpatelpcy@gmail.com

**Keywords:** green synthesis, chamomile nanocatalyst, dye degradation, catalytic reduction

## Abstract

The green chemistry method is the preferred approach for synthesizing metal and metal oxide nanoparticles because of its low toxicity, environmental friendliness, feasibility, and safety to human health compared with other chemical or physical methods. The present work reports the phytogenic synthesis of palladium nanoparticles (PdNPs) using an aqueous extract of *Matricaria recutita* (Chamomile). The phytochemical-mediated synthesis of PdNPs is an economical and eco-friendly approach without using toxic elements as reducing and capping or stabilizing agents. The UV-visible spectroscopic characterization was initially used to confirm the preparation of PdNPs using an aqueous extract of *M. recutita* flowers as a bioreductant for the reduction of Pd^2+^ to Pd^0^ without using any extra capping and reducing agents. The appearance of surface plasmon resonance (SPR) peak at 286 nm confirmed the formation of *M. recutita* extract-based PdNPs. Furthermore, the PdNPs were characterized by TEM, SEM, EDX, XRD, XPS, and FTIR to confirm their proper synthesis. The thermogravimetric analysis (TGA) was implemented to interpret the decomposition pattern and thermal stability of as-synthesized PdNPs. The biosynthesized PdNPs were further applied as a nanocatalyst in degradation of an azo dye Congo red (CR) in the presence of NaBH_4_. The catalytic reduction of 4-nitrophenol (4-NP) to 4-aminophenol (4-AP) was also investigated in the presence of NaBH_4_. All the catalytic reactions were performed in water, and no significant loss in catalytic activity was observed after recovery and reusability of the biosynthesized PdNPs.

## 1. Introduction

The eco-friendly approach of developing nanoparticles was implemented a few years ago to enhance the properties of such materials, possibly upon miniaturization [1]. The advancement in the technology of developing nanoparticles as compared to the earlier available facilities of probing nano-size is much radical [2,3]. As such, nanoparticles are applied for various applications upon their behavior of unique and specific features, such as smaller size, preciseness, enhanced surface area, and morphology [4,5]. In light of eco-friendliness of nanomaterials and economic yield, the biogenic routes are highly preferred for capping and fabrication, though seeking more and more attention by academic and industrial researchers [6,7,8]. The synthesis of nanoparticles is determined by three conditions: (i) choice of eco-friendly solvent medium, (ii) reducing agent, and (iii) nontoxic stabilizing agent [9,10]. Nanomaterials are long ago involved in providing solutions to different environmental and technological challenges by frolicking their role as a catalyst, and applied in the photo-degradation of dyes, wastewater treatment, medicine, and solar energy conversion [11,12,13,14,15]. Moreover, the nanomaterials fabricated from the biogenic origin, such as plants, bacteria, fungi, or any other biomass, had proven their potential application in serving science and technology [16,17,18,19,20]. The plant parts or their extracts are rich in phytochemicals with inherent antimicrobial properties [21]. Scientists concerned with nanotechnology are presently involved in the idea of using such phytochemicals as reducing agents in synthesizing metallic nanoparticles [22]. However, the nanoparticles are synthesized by various physical and chemical approaches, and do not resist to a couple of well-known methods, including microwave-assisted, heat evaporation, photochemical reduction, hydrothermal, electrochemical reduction, and so on [14,23,24,25,26]. The biogenic and/or green synthetic approach of nanoparticle synthesis is preferred over the traditional routes in terms of its eco-friendly, non-hazardous, cheap, simple, economical, and less harmful effects on the biosphere [27]. The biogenic synthesis upon bio-fabrication of metallic nanoparticles seeks immense attention being environment-friendly with mild experimental parameters such as pH, temperature, and pressure [28]. The green approach has been used to develop stable palladium nanoparticles (PdNPs) from different biomass sources. The bio-synthesized PdNPs have been reported from various species of bacteria, including *Shewanella oneidensis*, *Geobacter sulfurreducens* and *Plectonema boryanum* [29,30,31]. The peel extracts, including *Annona squamosal* and *Musa paradisiaca* (Banana) have also been used for the bio-synthesis of PdNPs [32,33].

The phytochemicals of various medicinal plants are sources of medicine after their inherited responses to prevent and treat many disorders. These bioactive components of medicinal plants are analogous with chemical compounds and are used to cure many diseases [34,35]. These phytochemicals or active chemical species are being involved as stabilizing, reducing, or capping agents in the biosynthesis of palladium nanoparticles [36,37]. The phytochemical composition of perennial herb chamomile (*Matricaria chamomilla*) of the Compositae family has been studied because of its various pharmacological responses as therapeutics for inflammations, ulcers, rheumatic pains, wounds, neuralgia, and as mouthwashes for treating gingivae [38,39]. The chamomile herb extract is worth accomplished as a reducing and stabilizing agent for the biosynthesis of metallic nanoparticles [40,41,42]. Moreover, the medicinal herb is rich in phytochemicals with active pharmacological compounds such as apigenin, luteolin, patuletin, quercetin, phenolics and flavonoids, and other components, including terpenoids, sesquiterpenes, flavonoids, phenylpropanoids (chlorogenic acid and caffeic acid), and coumarins (herniarin and umbelliferone) [43,44,45]. 

The management and the irradiation of water pollutants from industrial activity have exhibited a prompt increase in developing more effective bioreductants within the few decades [46]. Various approaches and techniques have been introduced to overwhelm such pollutants, but awkwardly ended up with the generation of secondary pollutants. So far, researchers have introduced an advanced oxidation process (AOP) for the fossilization of such organic pollutants, mostly the dyes molecules, by the generation of reactive species (such as ^•^OH radicals). In this regard, the fossilization of organic dyes, i.e., Congo red (CR), Methyl orange, Sunset yellow, and Tartrazine, has been irradiated as such azo dyes are well-known mostly as carcinogenic and mutagenic in nature. Meanwhile, the semiconductor metal oxides (SMOs) have been utilized as photocatalysts for incomplete fossilization of harmful organic pollutants into non-hazardous smaller degraded compounds (CO_2_ and H_2_O) [30,47]. In this research, we report the biosynthesis of PdNPs from *Matricaria recutita* flower extract and their utilization in the photochemical degradation of an azo dye, CR. The modified Williamson- Hall method accentuates the crystalline nature of biosynthesized PdNPs. In addition, the smaller size of nanoparticles, as observed from the TEM images, is the motivation behind using the PdNPs as a carrier of electrons in reducing the azo dye (CR) molecules in the presence of NaBH_4_ as a nanocatalyst. 

## 2. Materials and Methods

The flowers of *Matricaria recutita*, also known as German chamomile, were purchased from the local market in Jeddah, Saudi Arabia. Palladium chloride (PdCl_2_; (99.98%; molecular weight—177.33) was purchased from Sigma-Aldrich (USA) and used as a precursor for the biosynthesis of PdNPs. Congo red dye (C_32_H_22_N_6_Na_2_O_6_S_2_; molecular weight—696.66) and sodium borohydride (NaBH_4_; 98.0%; molecular weight—37.83) were also acquired from Sigma-Aldrich. All other chemical reagents were of analytical-grade and used as received, without any further purification. All stock solutions were prepared with ultrapure Milli-Q water.

### 2.1. Fabrication of PdNPs by Green Route

*Matricaria recutita* flowers were washed several times using double distilled water to eliminate impurities from the surface of the flowers. After completion of thorough washing, flowers were sun-dried and then crushed into powder. A 10 g of powdered form of *M. recutita* flowers was dispersed into a 250 mL Erlenmeyer flask containing 200 mL of deionized water. The Erlenmeyer flask was stirred on a magnetic stirrer at 50 °C for 30 min. The resulting extract was cooled down to room temperature followed by centrifugation at 6000 rpm for 5 min, and the supernatant was separated by filtration through a Whatman filter paper to collect a clear flower extract. The flower extract was stored at 4 °C for further preparation of PdNPs. PdNPs were prepared by mixing 50 mL of *M. recutita* flower extract with 50 mL of 0.01 M palladium chloride under continuous stirring at 50 °C for 2 h. The nanoparticle formation was observed by the consistent change in color from orange to dark brown. This dark brown solution was centrifuged at 2000 rpm for 30 min to separate the solid material, and the supernatant liquid was discarded. The material was washed several times with ethanol, double distilled water, and finally dried at 80 °C for 2 h in an oven.

### 2.2. Characterizations

The UV-visible spectroscopy using the Shimadzu UV-1280 spectrophotometer at 200–800 nm scanning range verified the biosynthesis of the PdNPs. Fourier-transform infrared spectroscopy (FTIR) (Compact FT-IR Spectrometer ALPHA II) was used to determine the role of biomolecules present in the *M. recutita* flower extract. Transmission electron microscopy (TEM) (JEM-3010, JEOL, Japan) was used to determine the shape, size, and particle size distribution of the as-synthesized PdNPs. For TEM analysis, the PdNPs suspension was drop-casted on the carbon-coated copper grid and allowed to dry overnight at room temperature. The scanning electron microscopy (SEM) (JEOL Model JSM—6390LV) analysis was performed by placing the powdered sample on the carbon stub using a sputter coater. The elemental composition of the as-prepared nanoparticles was performed by recording the energy dispersive X-ray (EDX) spectra on JEOL Model JED—2300. The purity and the crystallite size of the Pd NPs were determined by Rigaku X-ray diffractometer (model: ULTIMA IV, Rigaku, Japan) with a Cu Kp X-ray source (λ = 1.54056 Å) at 40 kV and current of 40 mA in the range of 20° to 90°. X-ray photoelectron spectroscopy (XPS) analysis was performed by using Thermo Scientific Escalab 250 Xi XPS instrument with Al Ka X-rays. Thermogravimetric analysis (TGA) was performed using Perkin Elmer STA-8000 at the heating rate of 10 °C per min under a nitrogen atmosphere.

### 2.3. Evaluation of the Catalytic Performance of PdNPs

To investigate the catalytic performance of the biosynthesized PdNPs, the degradation of CR dye and catalytic reduction of 4-NP to 4-AP in the presence of NaBH_4_ were chosen as model catalytic reactions. The catalytic experiments for CR dye degradation and 4-NP reduction by PdNPs were performed at 30 °C in the presence of excessive NaBH_4_ as compared to dye concentration. The progress of the degradation reactions was monitored by recording the UV-visible spectra at definite time intervals. The stock solutions of CR dye, 4-NP, and NaBH_4_ were prepared with high-purity water obtained from a Millipore Milli-Q system (resistivity > 18 MΩ cm at 25 °C). The blank experiments of CR dye and 4-NP were performed in the absence of PdNPs, and NaBH_4_. The λ_max_ values of CR dye and 4-NP were monitored at 498 nm and 318 nm, respectively. In a typical experiment, 3 mL of 0.5 × 10^−4^ M CR dye solution was mixed with 0.5 mL (1.0 × 10^−3^ M) freshly prepared NaBH_4_ solution, and finally, an amount of 4 mg of PdNPs was added to this reaction mixture as a catalyst. Under similar experimental conditions, 3 mL of 0.5 × 10^−4^ M 4-NP solution was mixed with 0.5 mL (1.0 × 10^−3^ M) freshly prepared NaBH_4_ solution followed by addition of 4 mg of PdNPs to this reaction mixture. The progress of the reaction was monitored by UV-visible spectroscopic measurements in the scanning range of 200–800 nm. It was clear from the continuing decolorization and decline in the λ_max_ of the CR dye solution that PdNPs played a significant role in the degradation process. The catalytic reduction of the 4-NP to 4-AP was also monitored by recording the time-dependent absorption UV-visible spectra of the reaction mixture at regular time intervals of 1 min. The following equation determined the catalytic efficiency of the PdNPs:

(1) $$Catalytic\;efficiency\;\left(\%\right)=\frac{C_{0}-C_{t}}{C_{0}}\;\times 100$$

where *C*_0_ is the initial CR concentration, *C_t_* is the CR concentration in the solution at a given time (*t*).

### 2.4. Reusability of PdNPs

The reusability of the catalyst after the completion of the first catalytic cycle was verified to confirm the stability of the biosynthesized PdNPs. In this study, PdNPs were recovered through centrifugation (10,000 rpm for 30 min) after completing the first cycle of CR dye degradation. The separated nanoparticles were thoroughly washed and applied for subsequent cycles to check the catalytic efficiency of as-prepared PdNPs under similar experimental conditions. The catalytic performance of the PdNPs was monitored through UV-visible spectroscopy as a function of time to evaluate the reduction kinetics for the reusability of the PdNPs.

## 3. Results and Discussion

**Figure 1:**
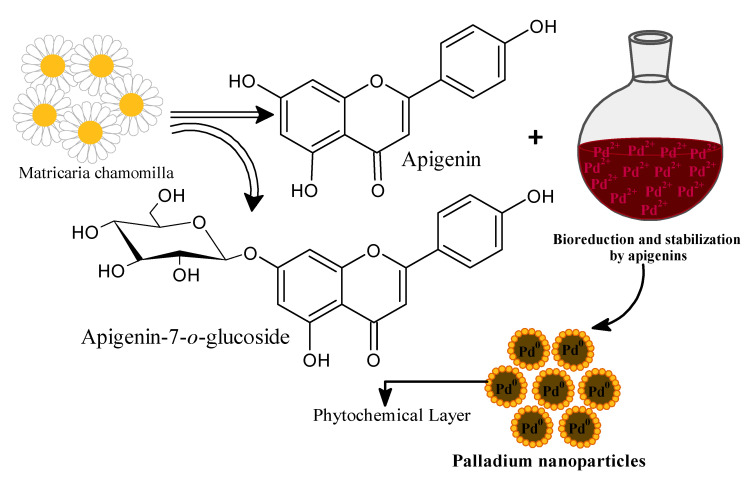
Bioreduction and stability of PdNPs using aqueous extract of *Matricaria recutita* flowers.

The PdNPs were synthesized using a simple in-situ method after treating the palladium chloride solution with aqueous flower extract of *M. recutita* at 50 °C. Since no external reducing agent was used in the reaction, it is reasonable to conclude that the phytochemical components were able to reduce Pd^2+^ to Pd^0^ and played a significant role in surface capping. It was proposed that the flower extract of *M. recutita* rich in various flavonoids, particularly apigenin and apigenin-7-o-glucoside, was responsible for the reduction of Pd^2+^ ions, thereby forming stable PdNPs (Figure 1). It was also observed that the transparent pale-yellow color of H_2_PdCl_4_ aqueous solution turned into the dark brown color on the addition of the flower extract of *M. recutita* after 2 h of reaction time. The physically observed dark brown color of the reaction mixture directs the bio-synthesis of PdNPs as accentuated from the excitation of surface plasmon vibrations [48]. The parameters such as ingredient ratio, reaction time, pH of the mixed solution, and temperature are essentially crucial in the metal reduction process [49]. The shape, size, aggregation state, and local environment, which exhibit different colors and optical properties of noble metal nanoparticles, directly influence the obtained spectral position and the width of the observed absorption band. Metal nanoparticles possess surface plasmon resonance (SPR) after the collective oscillation of conduction electrons in resonance with a wavelength of irradiated light. 

### 3.1. UV-Visible Spectroscopic Analysis of M. recutita Flower Extract and PdNPs

**Figure 2:**
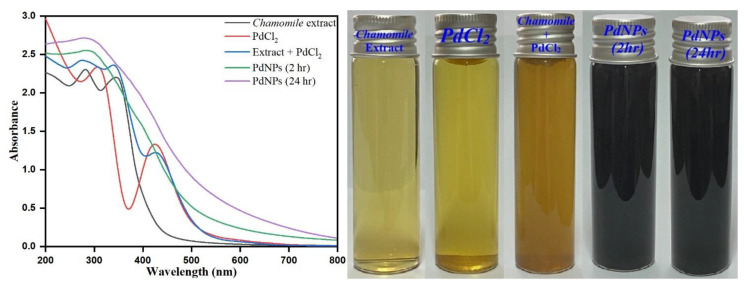
UV-visible spectrums of *chamomile extract*, PdCl_2_ solution, *Matricaria* recutita flower extract *+ PdCl_2_ reaction mixture, PdNPs at 2 h* and PdNPs at 24 h and their respective optical images.

First, the UV-visible spectroscopic analysis of *M. recutita* flower extract was performed to demonstrate the presence of polyphenolic compounds in the aqueous extract and their potential ability to reduce Pd^2+^ to Pd^0^. Further, optical properties of PdNPs were analyzed by a UV-visible spectrophotometer in the wavelength range of 200–800 nm. The observed intense peaks at ca. 268 and ca. 334 nm are attributed to benzoyl π→π* transitions associated with the presence of flavonoids, i.e., apigenin in *M. recutita* flower extract [50,51] as depicted in Figure 2. The apparent absorption peak for pale yellow solution of palladium was observed at 422 nm due to the presence of Pd(II) ions (Figure 2). After addition of *M. recutita* flower extract to the Pd(II) ion solution, visual monitoring revealed a progressive change in the color of the reaction medium from yellow to dark brown within 2 h of the reaction time. Further, the color of the reaction mixture changed from pale yellow to deep dark brown, which clearly indicated the formation of Pd(0) from Pd(II) ions reduced by the phytochemicals present in the aqueous extract of *M. recutita* flower, as also evident from the optical images shown in Figure 2. The typical absorption peak of Pd(II) ions completely disappeared, and a Pd(0) surface plasmon resonance (SPR) peak was observed, suggesting that Pd(II) was reduced to Pd(0) [52,53]. The absorption band about 422 nm caused by the presence of Pd(II) ions vanished, and a Pd(0) surface plasmon resonance (SPR) peak was observed, suggesting that Pd(II) was reduced to Pd(0). These findings reinforce the suggestions that phytochemicals, such as lavonoids, tannins, phenolics, and polyols, present in the flower extract of *M. recutita*, are thought to be the main driving force elements behind the reduction of metal (II) ions to metal (III) ions (0). Figure 2 also displays the absorption spectra of palladium colloidal suspension after 24 h of bioreduction by *M. recutita* flower extract, and no precipitation was observed, emphasizing the formation of stable PdNPs. The complete nucleation and the fabrication of biosynthesized PdNPs required a suitable reaction time.

### 3.2. FTIR Analysis of PdNPs

**Figure 3:**
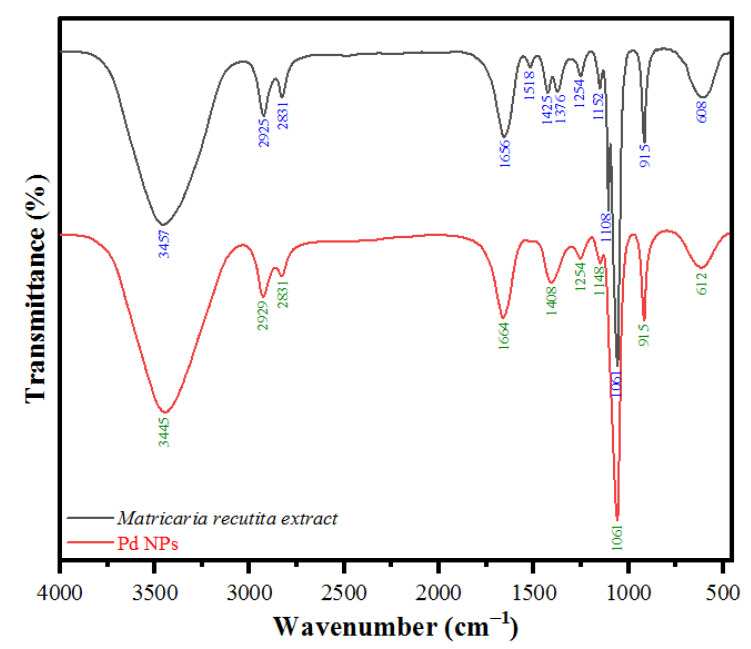
FT-IR spectra of PdNPs synthesized by using the flower aqueous extract of *Matricaria recutita***.**

FTIR spectroscopy analysis was used a valuable tool for understanding the role of biomolecules present in the *M. recutita* flower extract used in the formation of PdNPs and for investigating the chemical surroundings on the surface of the PdNPs (Figure 3). The FTIR analysis was endorsed to determine the presence of different functional groups responsible for the reduction of Pd(II) ions to PdNPs as shown in Figure 3. The presence of intense peaks emphasizes the role of altered functional groups of phytochemicals in the reduction process on to the surface of biosynthesized PdNPs. The PdNPs possessed most intensive peaks at 3445 cm^−1^ attributed to the presence of (–OH) groups of alcohols and phenols, 2929 cm^−1^ and 2831 cm^−1^ represented (–CH_2_) stretching vibrations, and 1664 cm^−1^ attributed to the presence of (C=O) stretching vibration, including conjugated ketones, aldehydes, quinones and esters. Peak at 1408 cm^−1^ corresponded to (C–N) stretching modes, whereas peak at 915 cm^−1^ attributed to the (=C–H) bending vibration of alkenes (Figure 3). However, the presence of similar FTIR peaks, such as 3457 cm^−1^, 2831 cm^−1^, 1656 cm^−1^, 1425 cm^−1^, 1376 cm^−1^, 1254 cm^−1^, and 915 cm^−1^ corresponded to various functional groups of phytochemical composition of *M. recutita* flower extract. Moreover, 1208 cm^−1^ represented to the presence of (–C–N) stretching vibration of aliphatic amines, 1254 cm^−1^ attributed to the presence of carboxylic groups (–COOH), 1148 cm^−1^ attributed to the presence of (C–O–C) stretching vibrations, and 1061 cm^−1^ attributed to (–C–N) stretching vibration of aliphatic amines, approximating the successful biosynthesis of PdNPs. In addition, the prominences of such peak intensities appeared after the flavanols, which are phenolic in nature, and as reported previously, phenolic molecules are being involved in the reduction process and stabilization of nanoparticles [54]. Moreover, it has also been reported that the presence of phenolic molecules, flavanols, amino acids, fatty acids, and vitamins is involved in the reduction process of PdCl_2_ to PdNPs [54,55,56]. The corresponding characteristics of the observed peaks in the FTIR analysis indicate the presence of phytochemicals in the synthesis and fabrication of PdNPs. The results inferred that Pd(II) reduction to Pd(0) occurred via the oxidation of hydroxyl to carbonyl groups. In addition, the flavonoids of *M. recutita* flower extract played their role as reducing agents and the amino groups as stabilizing agents in the reduction of Pd(II) to Pd(0) of biosynthesized PdNPs.

### 3.3. XRD Analysis of PdNPs

**Figure 4:**
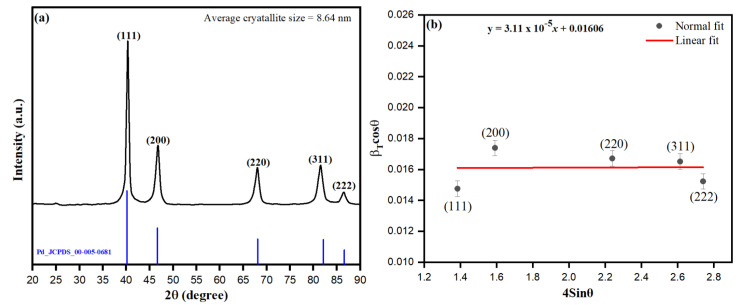
(**a**) XRD pattern for the PdNPs, (**b**) Williamson-Hall plot of PdNPs.

The as-biosynthesized PdNPs from *M. recutita* flower extract were determined from the discrete peaks observed at 2θ of 40.68, 54.44 and 65.48 with corresponding crystallographic planes at (111), (200) and (220) angles, respectively, referred to the pure face-centered cubic phases of biosynthesized palladium (JCPDS card no. 05-0681) (Figure 4a). From the observed 2θ peak intensities in Figure 4a, the attained positions and the width of peaks are associated with the nano-crystalline nature of PdNPs [57]. The observed unsplit peak at 2θ (40.68) indexed as (111) was prominently acting as the principle orientation peak of PdNPs with concurrent (200) and (220) peaks, which approximates the formation of small-sized PdNPs with high crystalline nature [58]. The average crystalline size (8.63 nm) of PdNPs was calculated in accordance with Scherrer’s method using the following equation:

(2) $$D=\frac{k\lambda }{\beta cos\theta }$$

where *D* represents the average crystalline size, *β* as the plane of full-width (111) at half maxima (FWHM) intensity, *λ* as X-ray wavelength (1.54 Å) of radiation, *θ* as Bragg’s angle (2θ). The average crystalline size as per the method was calculated to be 10.8 nm. Further, the crystalline size of PdNPs was accentuated by the modified Williamson-Hall method according to the equation:

(3) $$\beta cos\theta =\frac{k\lambda }{D}+4\varepsilon sin\theta$$

where *β* is the full width at half maxima or the peak broadening, which takes into account the particle size broadening and strain broadening [59], *D* = crystalline size, *k* = shape factor (0.9), *λ* = wavelength of Cukα radiation, and the Ɛ is the strain. Figure 4b shows the Williamson-Hall of β_hkl_cosθ verses 4sinθ. The linear fit gives the slope and y-intercept, from which the lattice strain and grain size were calculated. The following equations determined the dislocation density (*δ* nm^−2^) and micro-strain (*ε*), respectively:

(4) $$\delta =\frac{1}{D^{2}}$$

(5) $$\varepsilon =\frac{\beta }{4tan\theta }$$

The average crystalline size for PdNPs as per the method was calculated to be 8.63 nm. The diffraction data were used to estimate the crystallite size, d-spacing, dislocation density (*δ*), and microstrain (*ε*), as presented in Table 1.

### 3.4. Surface Analysis (TEM, SEM, EDX and XPS) of PdNPs

**Figure 5:**
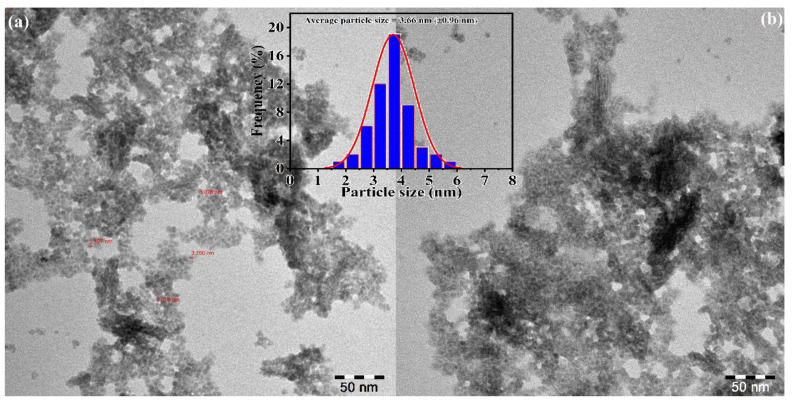
Transmission electron microscope (TEM) images (**a,b**) and particle size distribution histogram (*insert*) of PdNPs.

Transmission electron microscopy (TEM) analysis was implemented to further examine the understanding behind the surface morphology, dispersion, and diameter of biosynthesized PdNPs. The TEM images clearly showed that the PdNPs were spherical with narrow size distribution (Figure 5). The average particle size of the PdNPs calculated from the corresponding diameter distribution was approximately uniform with an average size of 3.66 nm (Figure 5 (*inset*)), which could be due to insufficient energy provided at the reaction temperature (50 °C) for the surface plasmon resonance peak of palladium nanoparticles. The nanoparticles prepared at 50 °C also had a small size and high dispersion, indicating complete reducing and stabilizing efficiency of *M. recutita* flower extract. The results of TEM studies revealed that PdNPs were stable with a slight tendency to aggregate, which could be due to the presence of phenols and flavonoids on the surface of PdNPs. It is believed that the phenols and flavonoids play a significant role in the stabilization/capping of PdNPs to inhibit the particle aggregation to some extent. 

**Figure 6:**
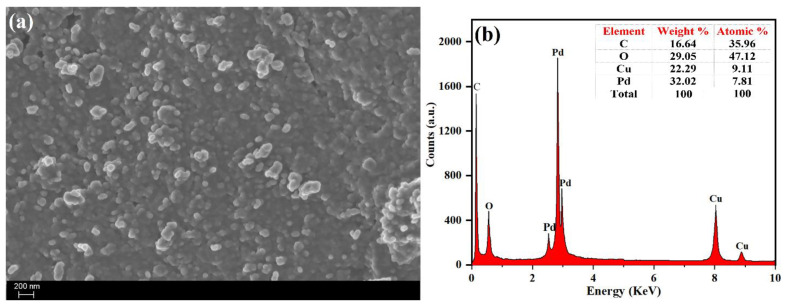
(**a**) Scanning electron microscopy (SEM) image, and (**b**) EDX spectrum of PdNPs.

It is apparent from the SEM image (Figure 6) that the PdNPs as-biosynthesized using *M. recutita* flower extract were spherical in morphology with narrow distribution and showed less agglomeration due to the presence of phytochemicals as capping agents on the surface of PdNPs. The elemental composition analysis of as-synthesized PdNPs was determined by the EDX analysis and the observed results indicate that the PdNPs synthesized were obtained with high purity. As shown in Figure 6b, the intensity peak around 3.0 keV endorsed the presence of metallic Pd, as reported in a previous study [60]. In addition, the EDX spectrum also indicated the presence of a few traces of carbon and oxygen, which could be attributed to the presence of organic capping molecules on the surface of the PdNPs, responsible for reduction and stabilization/capping of PdNPs [60]. The presence of copper signals belongs to the surface of copper grid used for the sample characterization.

**Figure 7:**
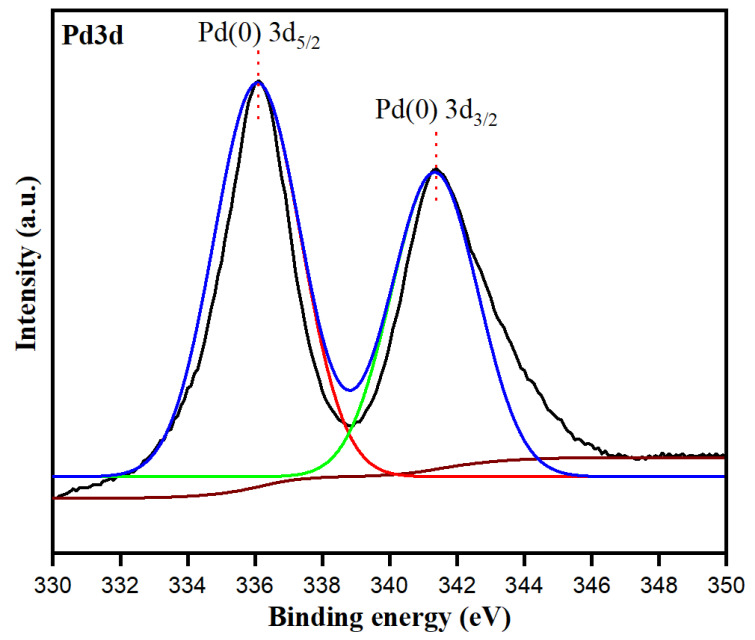
XPS spectrum showing the binding energy of Pd 3d.

X-ray photoelectron spectroscopy (XPS) is a surface-sensitive analytical technique that can be used to identify the elements present in the synthesized sample, thereby revealing its metallic state. Elemental composition of PdNPs measured by XPS was used to further study the formation characteristics of PdNPs. Figure 7 shows the high-resolution narrow scene (Pd 3d region-Pd 3d5/2 and Pd 3d3/2) of as-synthesized PdNPs. The binding energy peaks at 335.4 eV and 340.5 eV corresponded to the spin-orbit splitting components, Pd 3d5/2 and Pd 3d3/2, respectively. The observed binding energy values for Pd 3d coincided with the reported values of Pd(0) [61], which confirms the successful biosynthesis of PdNPs in the zero-oxidation state using *M. recutita* flower extract.

### 3.5. Thermal Gravimetric Analysis (TGA) and Differential Thermal Analysis (DTA)

**Figure 8:**
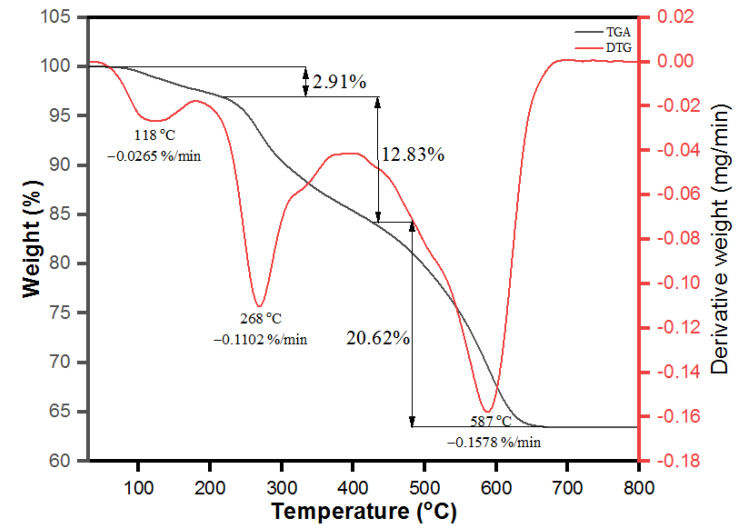
Thermal gravimetric analysis (TGA) and differential thermal analysis (DTA) of PdNPs.

The thermogravimetric analysis (TGA) was attained to accentuate the thermal stability of PdNPs biosynthesized from *M. recutita* flower extract as depicted in Figure 8 (Black line). The percentage of weight loss during thermal decomposition of nanoparticles was estimated. The analysis was operated under a nitrogen atmosphere up to 800 °C at a linear heating rate of 10 °C per min. Further, the close perusal of Figure 8 emphasizes that the biosynthesized PdNPs attained about total of 36.36% weight loss up to 650 °C. The observed initial weight loss of 2.91% was observed in the differential thermal analysis (TGA) from 50–210 °C, which was endorsed from the evaporation of moisture content as adsorbed from the sample by the surroundings and capping of volatile phytochemicals onto the surface of PdNPs [62]. The presence of peaks at 118 °C with derivative weight % rate at 0.0265 %/min and at 268 °C with derivative weight % rate at 0.1102 %/min analyzed by DTG analysis were attributed to moisture evaporation and thermal decomposition of volatile phytochemicals of organic aromatic rings onto the surface of biosynthesized PdNPs. The second weight loss from 210 °C up to 420 °C is believed from the thermal degradation of phytochemicals such as proteins, phenolics, and flavonoids of *M. recutita* flower extract capped as stabilizing agents onto the surface of PdNPs as analyzed by the TGA analysis with 12.83% of weight loss [62]. Further weight loss from 420 °C onwards occurred upon the thermal degradation of remaining carbon residues of phytochemicals onto the surface of PdNPs, as supported by DTG peak observed at 587 °C with derivative weight % rate at 0.015 %/min and weight loss (~20.62%) calculated from TGA analysis. The observed results illustrate that the biosynthesized PdNPs have higher thermal stability. 

### 3.6. Catalytic Dye Degradation of Congo Red by PdNPs

**Figure 9:**
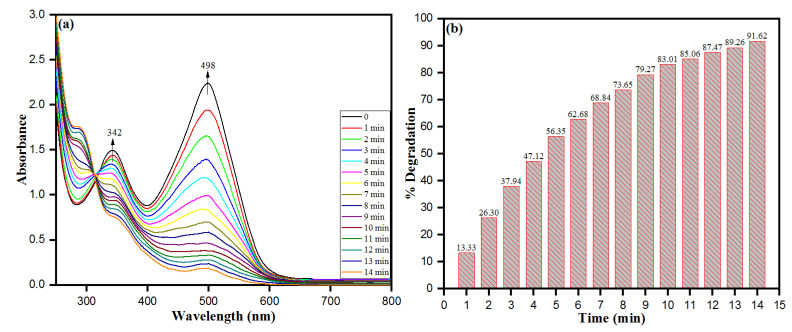
(**a**) UV–visible spectra of CR degradation under optimum experimental conditions; (**b**) percentage degradation of CR dye with respect to irradiation time. (Experimental conditions: (CR) = 0.5 × 10^−4^ M, (NaBH_4_) = 1.0 × 10^−3^ M, PdNPs = 4 mg, Temperature = 30 °C).

The environmental protection and remediation upon reduction of azo dyes are essential concerns of wastewater treatment. The biosynthesized PdNPs from the *M. recutita* flower extract are anticipated behind the photodegradation of an azo dye (Congo red) based on their high surface area to volume ratio in a dose-dependent manner. Here, we used Congo red (CR) an azo dye with molecular formula C_32_H_22_N_6_Na_2_O_6_S_2_ being toxic and non-biodegradable, primarily eradicated from dyeing industries for degradation. In UV-visible spectroscopy, the dye CR displayed an intensity peak at ca. 496 nm. The sodium borohydride (NaBH_4_) alone was not tested for degradation of dye molecules as the dye degradation in the presence of sodium borohydride was favorable in the thermodynamic standard point but unfavorable kinetically after the huge difference in the reduction potential of electron-donating sodium hydride and electron acceptor Congo red dye. The biosynthesized PdNPs were added to the reaction mixture solution as they provide a high surface area to the volume ratio with an increased reduction rate of the reaction. The presence of nanocatalyst to the reaction mixture helps in shuttling electron (providing passage of electron to acceptor from the donor). The biosynthesized PdNPs act as a nanocatalyst and NaBH_4_ as an electron donor in the process of degradation of CR dye molecules. Our results emphasize that the CR dye molecules were reduced completely within 14 min as inferred by the disappearance of intensity peaks with increasing time intervals at ca. 496 nm (Figure 9a). The degradation efficiency of PdNPs to CR was calculated by degradation (%) efficacy expression (Equation (1): the (%) degradation of PdNPs to CR was approximately 91.62% as depicted in Figure 9b. The CR degradation was dependent on factors, such as the size and surface area of the nanocatalyst. In addition, the concentration of dye and the amount of NaBH_4_ used to hydrate ions in the electron transfer are two essential factors of dye remediation. In addition, a research reported CR reduction efficiency by 95.32% using PdNPs biosynthesized from biogenic sources such as cotton bolls peel [36]. The biosynthesized PdNPs of *M. recutita* flower extract and azo dye (CR) displayed higher redox potential differences. The potential differences were reduced significantly in the presence of NaBH_4_ (as an electron donor) and CR (as an electron acceptor), including PdNPs by reducing the activation energy barrier with a favorable kinetic process leading to the rapid reduction of the dye. The core concept behind the reduction process was that the PdNPs basically played a role as a carrier molecule or helped electron shuttling, thus facilitating the passage of electrons from donor NaBH_4_ to acceptor CR molecules in a reaction mixture [63].

**Figure 10:**
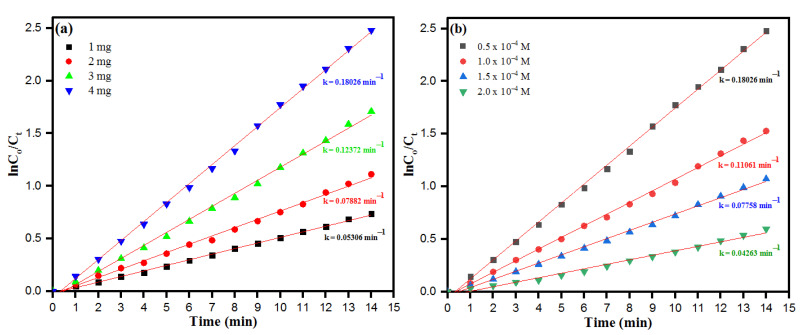
Plot of ln*C*_0_/*C_t_* vs. time for the reduction of CR as of function of (**a**) catalyst dosage and (**b**) initial CR dye concentration.

The close glance of Figure 10a,b showed a linear relationship between ln(*C*_o_/*C*) against irradiation time (min) for CR. The catalytic reduction of CR thus was determined by following the Pseudo-fist order kinetic as per the expression:

(6) $$\ln\left(C\right)=kt+\ln\left(C_{0}\right)$$

where *C*_0_ and *C* represent the initial concentration and the concentration after reduction time “*t*” of CR, respectively and *k* as the rate constant. The rate constant was calculated as per Equation (6) and was found to be 0.18062 min^−1^. The overall results of catalytic degradation of azo dye accentuate during the process, biosynthesized PdNPs of *M. recutita* flower extract act as a carrier of electrons, which helps the NaBH_4_ molecules in cleaving the azo bond of CR to reduce into nontoxic smaller fragments. Moreover, the degradation of CR and the formation of non-hazardous and less toxic molecules were determined from the obtained absorption intensities of CR (Figure 9a), which further vanished with specified time interval.

The pseudo-first-order kinetic analysis was deduced to determine the azo-dye degradation over biosynthesized PdNPs to NaBH_4_ with different catalytic doses from 1–4 mg at 30 °C, keeping (CR) = 0.5 × 10^−4^ M and (NaBH_4_) = 1.0 × 10^−3^ M constant as depicted in Figure 10a. The observed kinetic rate constant (k) decreased linearly with the increase of catalyst concentration. The results showed an increase in the catalytic concentration, and the decreased in the dye degradation upon the catalytic dosing behind the depletion of active sites of the catalyst. Moreover, the effect of the initial concentration of the dye was evaluated from the concentration range of (0.5–2) × 10^−4^ M (Figure 10b). The concentration of NaBH_4_ in the reaction mixture solution was higher in comparison to the concentration of CR, thus expecting the occurrence of the reaction as per pseudo-first-order kinetics. However, the rate (−d(A)/dt) of the reaction was attained from the linear steep part of the absorbance-time curve. The observed decreased trend of reaction rates with the high concentration of dye can be inferred after receiving a limited number of dye molecules by the catalyst to degrade. The rate of reactions was calculated as per Equation (4), for 0.5 × 10^−4^, 1.0 × 10^−4^, 1.5 × 10^−4^, and 2.0 × 10^−4^M to be 0.18062, 0.11061, 0.07758 and 0.04263 m^−1^, respectively. These results could be obtained when the remaining dye molecules in the bulk solution reach the surface of NaBH_4_ from the surface of carrier PdNPs until the earlier attached molecules are degraded. The ongoing process of dye degradation with depletion of the active sites of the catalyst with increased time intervals resulted in a decreased reaction rate. The decreases in reaction rate with higher concentrations are well-understood in support of competition created between reaction products and free dye molecules, or reaction intermediates to fasten them on the active sites of the catalytic surface. Interference of reaction intermediates or reaction products led to the poisoning of catalytic surface with corresponding delay in dye degradation with increase in both time interval and dye concentration. The onset of higher concentrations leads to more and more CR molecules adsorbing onto the surface of the catalyst, while with lack of direct contact of active sites results in the inhibitive effects on the dye degradation [64,65]. The rate of dye degradation increases at neutral pH because of the generation of –OH molecules helping in the reduction process. Furthermore, at alkaline pH, the rate of dye molecule degradation decreases because of the formation of Pd-hydro complexes rather than the nanoparticles.

**Figure 11:**
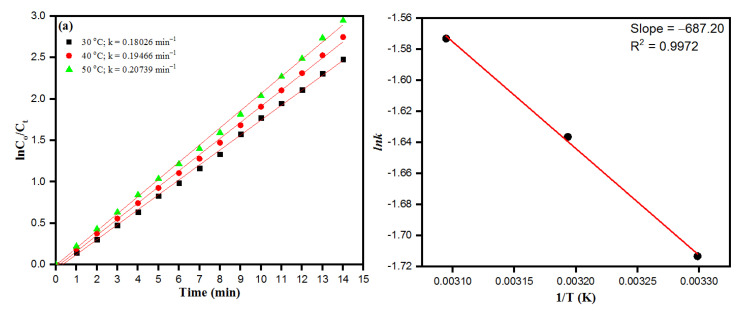
(**a**) Plot of ln*C*_0_/*C_t_* vs. time for the reduction of CR as of function of temperature. (**b**) Arrhenius plot for the calculation of activation energy (Ea). (Experimental conditions: (CR) = 0.5 × 10^−4^ M, (NaBH_4_) = 1.0 × 10^−3^ M, PdNPs = 4 mg).

The Arrhenius activation energy relation is the temperature-dependent kinetic equation used in chemical reactions, the equation is applicable in catalytic dye degradation reactions and to determine the rate constant and its relationship with changing temperatures. The kinetic plot of PdNPs at varying temperatures from 30 °C to 50 °C is depicted in Figure 11a. The obtained linear relation of ln(*C*_0_/*C_t_*) versus reaction time (*t*) is in accordance with the conventional Arrhenius temperature-dependent relation. As such, the activation energy plot as depicted in Figure 11b related to the chemical reduction of CR dye molecules in the presence of PdNPs as a catalyst was calculated following the Arrhenius equation:

(7) $$lnk_{app}=lnA-\frac{E_{a}}{RT}$$

where *E_a_* is the apparent activation energy, *R* as ideal gas constant 8.314 J K^−1^mol^−1^ and *T* is the absolute temperature. Moreover, the activation energy (*E_a_*) was calculated to be 5.71 KJ/mol. In general, relative lower activation energy was after the enhanced catalytic activity of biosynthesized nanoparticles serving as catalysts. The obtained lower values of *E_a_* suggest that the biosynthesized PdNPs as a biocatalyst can greatly lower the energy barrier for reduction of CR dye molecules.

The biogenic PdNPs in aqueous extract of *M. recutita* flowers are developed possible by a two-step mechanism. In step I, the complex formation of Pd(II) with carbonyl and hydroxyl functional groups of various phytochemical takes place in an aqueous *M. recutita* flower extract. The hydroxyl groups being antioxidant in nature with high reducing efficacy, are involved in the reduction of Pd(II) to Pd(0) atoms while the carbonyl groups undergo oxidation process. In step II, the stabilization of as-synthesized PdNPs was achieved by the surface capping of phytochemicals onto the surface of PdNPs via binding interaction of metal atoms with functional groups containing oxygen. The hypothesis was supported by the FTIR analysis of *M. recutita* flower extract. The decreased intensity peaks of hydroxyl and carbonyl functional groups along with the new peaks corresponding to carboxyl compounds emphasize the role of hydroxyl and carbonyl groups in both the synthesis and the stabilization of PdNPs [66].

**Figure 12:**
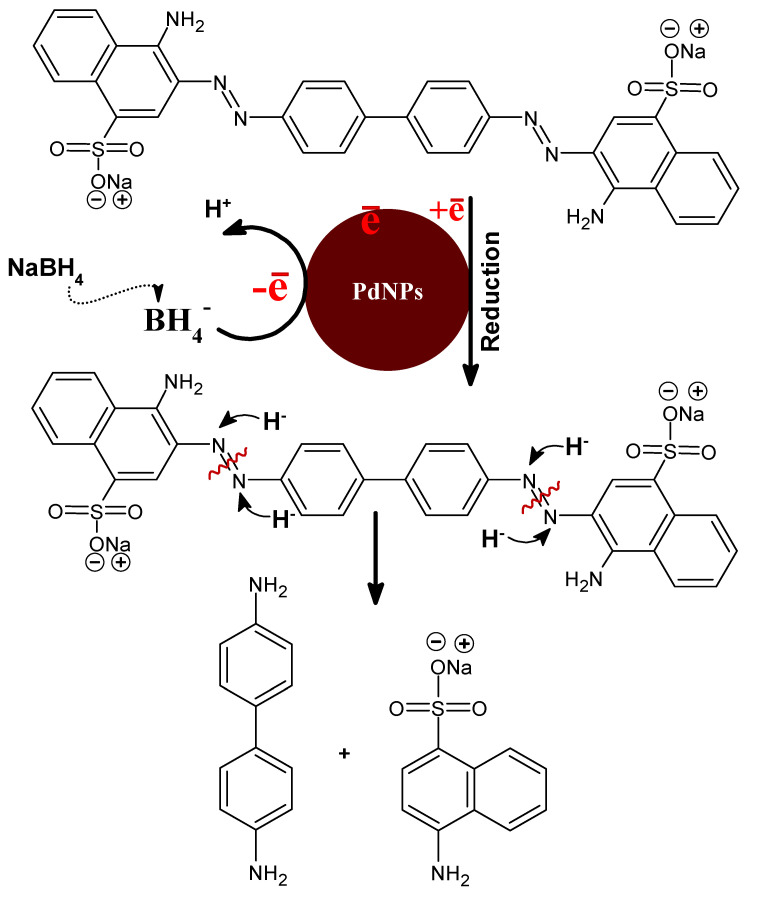
A plausible mechanism for the CR dye degradation by NaBH_4_ using PdNPs as a catalyst.

Figure 12 demonstrates the systematic representation after the possible mechanism of catalytic reduction of CR dye molecules by PdNPs in the presence of NaBH_4_ molecules [36,67]. The PdNPs as a catalyst played their role in the electron transfer as a carrier, and triggered the hydride ions to achieve the cleavage of azo-dye (CR) molecules so as to reduce into small nontoxic fragments. NaBH_4_ molecules alone were not efficient in reducing the CR dye molecules into small nontoxic fragments. Besides, addition of PdNPs as a catalyst to the reaction mixture played a significant role in the process of electron-shuttling as a mediator, i.e., electrons accelerated from NaBH_4_ via PdNPs-mediated hydride ions involved in the process of cleavage of dye molecules to nontoxic species. Ultimately, the dye degradation was physically accentuated from the disappearance of absorption intensities within the specified time intervals.

### 3.7. Catalytic Reduction of 4-NP over PdNPs

**Figure 13:**
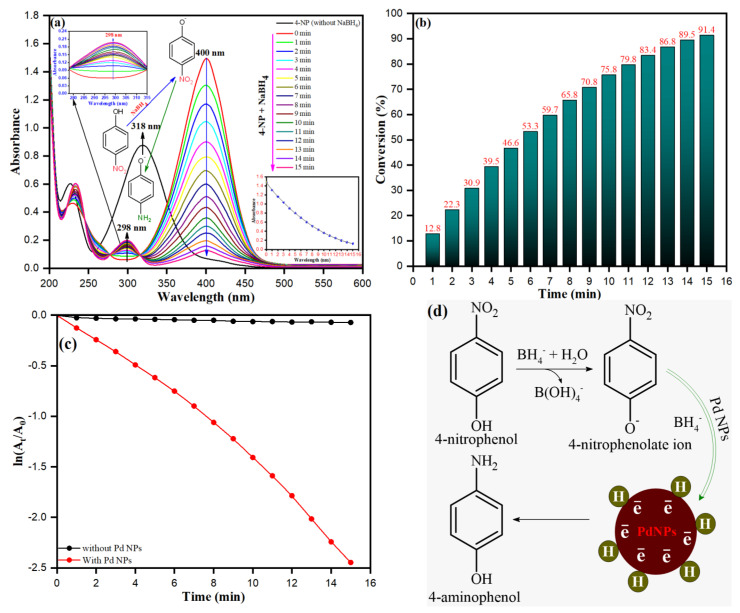
(**a**) The UV–vis absorption spectrum of 4-NP, (**b**) Conversion percentage of 4-NP to 4-AP, (**c**) Kinetic plot for the reduction of 4-NP into 4-AP, and (**d**) Possible conversion mechanism of 4-NP into 4-AP in the presence of PdNPs. (Experimental conditions: (4-NP) = 0.5 × 10^−4^ M, (NaBH_4_) = 1.0 × 10^−3^ M, PdNPs = 4 mg, Temperature = 30 °C).

We also tested the catalytic activity of PdNPs for the reduction of 4-nitrophenol (4-NP) in the presence of sodium borohydride (NaBH_4_). The UV-vis spectroscopy was used to test the catalytic reduction of 4-NP, as shown in Figure 13a. When a freshly prepared aqueous solution of NaBH_4_ was added, an absorption peak of 4-NP undergoes a redshift from 318 to 400 nm, leading to a dramatic change in solution color from light yellow to yellow-green due to the formation of 4-nitrophenolate ion. The absorption peak at 400 nm remained unchanged in the absence of a catalyst for a long time, suggesting that NaBH_4_ cannot reduce 4-nitrophenolate ion without a catalyst. Under ambient reaction conditions, the 4-NP was easily reduced to 4-AP in the presence of PdNPs and NaBH_4_, and the reduction reaction was completed in a much shorter time, as shown in the Figure 13a. The strength of the absorption peak of 4-NP at 400 nm decreased gradually with time, as shown in Figure 13a, and it nearly disappeared after 15 min, suggesting that all the 4-NP was converted to 4-AP. Meanwhile, a new absorption peak emerged at 298 nm, which grew in intensity over time. The normal absorption of 4-aminophenol was responsible for this new high (4-AP), suggesting that the catalytic reduction of 4-NP only created 4-AP. Figure 13b shows the conversion rate as a function of time, suggesting that approximately 91.4% conversion occurred after 15 min of reaction time. In this case, together with the UV-visible absorption data as demonstrated in Figure 13c, pseudo-first-order kinetics may be used to calculate the reaction rate of the current catalytic reaction. A linear plot of ln(*C_t_*/*C*_0_) versus reduction time in minutes may be used to calculate the rate constant (k) (Figure 13c). The rate constant (k) was calculated using the slope of linear plots of ln(*C*_0_/*C*) versus time (min) and found to be 0.16145 min^−1^. Good linearity between ln(*C*_0_/*C_t_*) and (*t*), and high values of the correlation coefficient R^2^ (0.986) demonstrated that the decoloration process perfectly fit to the first-order kinetics. The mechanistic pathway of p-nitrophenol reduction involves several steps. Figure 13d depicts the possible catalytic mechanism for reducing 4-NP to 4-AP in the presence of NaBH_4_ (d). Since PdNPs are used for catalytic reduction, BH_4_ and 4-NP are first diffused to the Pd surface from aqueous solution, and then the PdNPs act as catalysts, moving electrons from BH_4_ to nitrophenol. The excess borohydride BH_4_ ions from NaBH_4_ adsorbed on the surface of PdNPs and then transferred a hydride to the nitrophenol. The 4-nitrophenolate is reduced to 4-AP due to the thermodynamically unstable hydrogen atoms interacting with 4-nitrophenolate during the hydrogenation process.

### 3.8. Catalytic Recyclability of PdNPs

**Figure 14:**
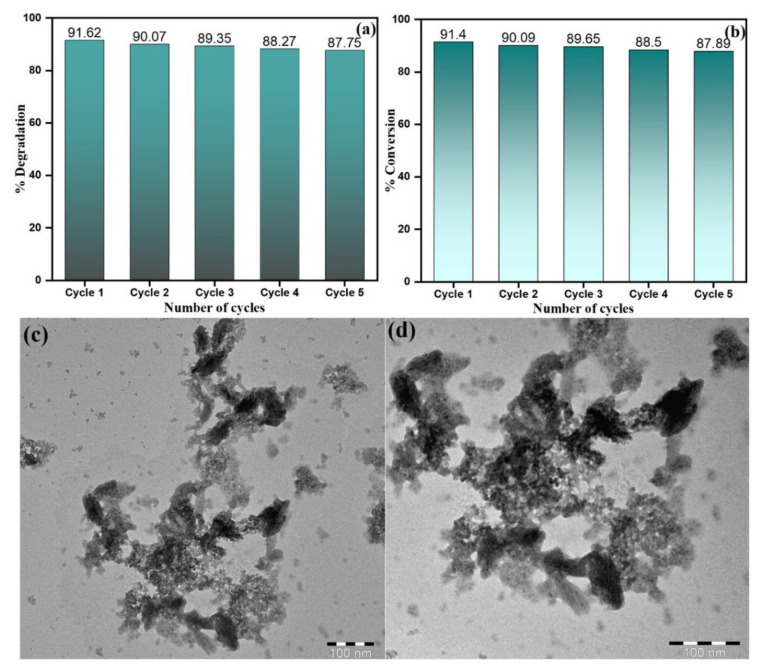
Catalytic recyclability of PdNPs for (**a**) CR dye degradation, (**b**) catalytic reduction of 4-NP. TEM image after five cycles of use for (**c**) CR dye degradation and (**d**) catalytic reduction of 4-NP.

The catalytic recyclability of PdNPs for CR dye degradation ((Experimental conditions: (CR) = 0.5 × 10^−4^ M, (NaBH_4_) = 1.0 × 10^−3^ M, PdNPs = 4 mg, Temperature = 30 °C) and catalytic reduction of 4-NP into 4-AP ((Experimental conditions: (4-NP) = 0.5 × 10^−4^ M, (NaBH_4_) = 1.0 × 10^−3^ M, PdNPs = 4 mg, Temperature = 30 °C)) was evaluated as depicted in Figure 14a,b, respectively. The recyclability evaluation of PdNPs upon CR dye degradation and catalytic reduction of 4-NP was comprehended by 5 cycles so as to infer stability and reusability of PdNPs. In visible light, keeping stability and recyclability in consideration upon each cycle, the PdNPs as a catalyst for CR dye degradation and catalytic reduction of 4-NP to -AP were estimated. After each cycle, the sample was centrifuged to isolate the catalyst from the solution, followed by washing with deionized water and dried over 100 °C for an hour. The obtained nanoparticles with their natural physical appearance after washing and drying were used for subsequent cycle treatment. Besides, it was apparently deduced from Figure 14a,b, the PdNPs as a nanocatalyst showed a slight decrease in CR degradation and 4-NP reduction with a minor loss after the consecutive cycles of degradation cycling experiment. After five cycles of reuse, the PdNPs were recovered and appropriately washed, and analyzed by TEM, as shown in Figure 14c,d. The TEM image of PdNPs (nanocatalyst) did not show any significant change in the morphology but showed approximately 4% decline in the catalytic activity after five cycles of reuse with little agglomeration. The catalytic recyclability experiments infer that the PdNPs as a nanocatalyst are worth applicable and suitable for catalytic application with appraising performance and stability.

## 4. Conclusions

The present work emphasizes the biosynthesis of palladium nanoparticles (PdNPs) by using the aqueous extract of *Matricaria recutita* flowers employing an eco-friendly green approach. *M. recutita* flower extract was successfully employed as a reducing and/or capping agent for the preparation of PdNPs without any addition of supportive chemicals. Formation and the morphology of as-synthesized PdNPs were confirmed by using various standard techniques, including UV-vis, FTIR, XRD, TEM, and EDX. In addition, the PdNPs were thermally stable with weight loss of fabricated phytochemicals onto their surface as observed upon TGA-DTG analysis. The highly crystalline and spherical PdNPs were used for the catalytic reduction of an azo-dye CR with approximately 92% degradation efficiency within 14 min along with high stability after 5 cycles of reuse as a catalyst. The as prepared PdNPs shows efficient catalytic reduction of 4-NP to 4-AP with excel-lent stability and reusability. The overall results highlight that this study provides a suitable platform for the effective and successful Green Synthesis of noble metal nanoparticles for their potential catalytic activities.

## Figures and Tables

**Table 1:** XRD parameters, including d-spacing, crystallite size, dislocation density, and micro-strain of PdNPs.

Peak Position 2θ (Degree)	hkl	β_T_ FWHM	d-Spacing (Å)	Crystallite Size d (nm)	Dislocation Density δ × 10^−3^ (nm^−2^)	Micro-Strain ε × 10^−3^
40.47322	111	0.90099	2.226958414	9.397367625	11.32367944	10.66395747
46.86824	200	1.08615	1.936914623	7.971694654	15.73615735	10.93383049
68.05806	220	1.15548	1.376487262	8.295964625	14.53002016	7.466512887
81.58613	311	1.24973	1.17903848	8.396541304	14.18401372	6.318879368
86.46536	222	1.19785	1.124586654	9.102831599	12.06832461	5.559412903
